# Translation and validation of the Chinese version of the Pancreatic Exocrine Insufficiency Questionnaire in patients after pancreatectomy: A cross-sectional psychometric study

**DOI:** 10.1097/MD.0000000000050171

**Published:** 2026-08-28

**Authors:** Huiping Yu, Wang Su, Lei Cui, Jianjian Wang, Kuirong Jiang, Xiaoping Fang

**Affiliations:** a Department of General Surgery, Pancreas Center, The First Affiliated Hospital with Nanjing Medical University, Nanjing, Jiangsu, China; b Office of Science, Nanjing Medical University, Nanjing, Jiangsu, China.

**Keywords:** cross-sectional study, pancreatectomy, pancreatic cancer, pancreatic exocrine insufficiency, patient-reported outcome, reliability, validity

## Abstract

Pancreatic exocrine insufficiency (PEI) is common after pancreatic resection, but no Simplified Chinese PEI-specific patient-reported measure has undergone published cross-cultural evaluation. We translated and adapted the Pancreatic Exocrine Insufficiency Questionnaire (PEI-Q) and evaluated selected psychometric properties in patients after pancreatectomy. Following Beaton guidelines, the PEI-Q was translated and cross-culturally adapted. This single-center cross-sectional study included 163 patients. Item analysis, content validity assessment, principal component analysis, principal axis factoring sensitivity analysis, parallel analysis, and confirmatory factor analysis of the original 3-domain structure were performed. Internal consistency was evaluated; test–retest reliability, criterion validity, and responsiveness were not. Overall Cronbach alpha was 0.813 (95% confidence interval: 0.742–0.857); domain alphas were 0.667, 0.719, and 0.603, with 2 domains below 0.70. Total-scale McDonald omega was 0.820. Corrected item-total correlations ranged from −0.006 to 0.680; removing item 14 increased alpha to 0.843. Exploratory analyses retained 3 factors, but confirmatory factor analysis did not support the original structure (comparative fit index, 0.710; Tucker-Lewis index, 0.664; root mean square error of approximation, 0.130; standardized root mean square residual, 0.130). Principal axis factoring confirmed low communality for item 14 (0.013). The bowel movement symptoms domain showed a 25.8% floor effect; no ceiling effects occurred. The Chinese PEI-Q showed strong content validity and acceptable overall internal consistency. However, 2 domains had borderline consistency, and the original structure was not confirmed. It may support symptom monitoring and patient-clinician communication but should not be used for diagnosis or regarded as fully validated until further evaluation.

## 1. Introduction

Pancreatic cancer accounted for approximately 495,773 new cases worldwide in 2020.^[1]^ Surgical resection, including partial or total pancreatectomy, remains an important treatment option for resectable pancreatic cancer.^[2]^ Because the pancreas has both endocrine and exocrine functions, loss of pancreatic tissue and reconstruction of the gastrointestinal tract may impair glucose regulation and digestive enzyme secretion.^[3]^ Pancreatectomy is therefore an important cause of pancreatic exocrine insufficiency (PEI), although PEI may also occur in chronic pancreatitis, cystic fibrosis, acute pancreatitis, and unresectable pancreatic cancer.^[4–7]^

PEI after pancreatectomy refers to inadequate synthesis or delivery of pancreatic enzymes to maintain normal digestion after pancreatic tissue resection and altered anatomy.^[8–10]^ Reported rates vary widely across surgical procedures and diagnostic methods. A systematic review reported PEI rates ranging from 36% to 100% after pancreatic or periampullary cancer surgery, and rates of 67% to 80% after distal pancreatectomy.^[6]^ In 1 study, all 26 patients had PEI 52 weeks after pancreatoduodenectomy.^[11]^ PEI-related symptoms include steatorrhea, abdominal pain, bloating, flatulence, loss of appetite, nausea, vomiting, and changes in stool characteristics.^[12–14]^ These symptoms may contribute to malnutrition, weight loss, postoperative complications, longer hospital stays, higher medical costs, impaired quality of life, and psychological distress.^[9,10,15–20]^ Postoperative assessment also intersects with longer-term PEI follow-up and nutritional management after pancreatic surgery.^[21,22]^

Patient-reported symptom instruments can therefore provide useful adjunctive information, especially when biochemical testing is not routinely accessible. Patient-reported outcome measures are increasingly incorporated into postoperative care because they capture symptom burden, functional impact, and treatment priorities that may not be apparent from routine clinical observations, while supporting patient-provider communication and longitudinal monitoring.^[18,23]^ In post-pancreatectomy care, a brief symptom measure may complement but should not replace objective testing when objective testing is available.

The Pancreatic Exocrine Insufficiency Questionnaire (PEI-Q), developed by Johnson et al, is a patient-reported outcome instrument designed to assess PEI-related symptoms and impacts.^[23]^ A subsequent psychometric evaluation supported its reliability and validity in PEI populations.^[18]^ To our knowledge, no simplified Chinese PEI-specific instrument with a published translation and psychometric evaluation was available before this study. Cross-cultural adaptation requires more than literal translation; it should preserve semantic, idiomatic, experiential, and conceptual equivalence so that the instrument represents the same underlying content in the target population.^[24]^ The PEI-Q has not previously been evaluated among Chinese-speaking patients after pancreatectomy. We focused on post-pancreatectomy patients because this group has a high risk of PEI, requires regular postoperative follow-up, and may particularly benefit from a concise symptom assessment instrument that facilitates communication between patients and healthcare providers. Accordingly, this study aimed to translate and cross-culturally adapt the PEI-Q into simplified Chinese and to evaluate selected psychometric properties among patients after pancreatectomy.

## 2. Methods

### 2.1. Study design and reporting

Following Beaton et al guidelines, the PEI-Q was translated and cross-culturally adapted from English into simplified Chinese.^[24]^ A single-center cross-sectional psychometric study was then conducted to evaluate the Chinese version. This study was reported with reference to relevant items of the Strengthening the Reporting of Observational Studies in Epidemiology statement. Because this was a psychometric study, results were interpreted as evidence about translation quality and selected measurement properties, not as evidence of diagnostic accuracy.

### 2.2. Ethical considerations

Ethical approval was granted by the Ethics Committee of the First Affiliated Hospital with Nanjing Medical University (approval no. 2022-SR-222). All participants were informed about the purpose and procedures of the study and provided written informed consent before participation. The study was conducted in accordance with the Declaration of Helsinki and the ethical principles of the Belmont report.

### 2.3. Original English instrument

The PEI-Q consists of 3 domains and 18 items. The 1st domain contains 7 items assessing abdominal symptoms, the 2nd contains 6 items assessing bowel movement symptoms, and the 3rd contains 5 items assessing PEI-related impacts. All items refer to the respondent’s experience during the past 7 days and are rated on a 5-point Likert scale from 0 to 4, indicating symptom frequency, severity, or impact. The scores of the abdominal symptom and bowel movement symptom domains are averaged to produce the total symptom score, which may support symptom assessment and clinical communication. The impact domain is completed only when respondents have been diagnosed with PEI. The scoring rules are shown in Table 1.

**Table 1:** Scoring rules of the PEI-Q.

Domain score	Formula
Abdominal symptoms (A)	A = sum of scores for items 1–7/7
Bowel movement symptoms (B)	B = sum of scores for items 8–13/6
Total symptom score	(A + B)/2
Impacts (C)	C = sum of scores for items 14–18/5
Total summary score (PEI patients only)	(A + B + C)/ 3

A = abdominal symptoms domain mean, B = bowel movement symptoms domain mean, C = impacts domain mean, PEI = pancreatic exocrine insufficiency, PEI-Q = Pancreatic Exocrine Insufficiency Questionnaire.

### 2.4. Cross-cultural adaptation process

Before cross-cultural adaptation, authorization was obtained from the original author by email. The translation and adaptation process followed the 5-stage approach proposed by Beaton et al.^[24]^

Stage 1: Three native Chinese speakers independently translated the English PEI-Q into Chinese. Stage 2: The translators and research team compared the 3 forward translations, discussed discrepancies, and produced a synthesized version by consensus. Stage 3: Two native English speakers independently back-translated the synthesized Chinese version without access to the original questionnaire.

Stage 4: An expert committee comprising all translators, the research team, a pancreatic surgeon, and a methodologist reviewed semantic, idiomatic, experiential, and conceptual equivalence. Particular attention was paid to patient-friendly Chinese expressions for stool characteristics, bowel movement frequency, and avoidance of fatty food because these concepts may be interpreted differently across educational and regional backgrounds. No item meaning was intentionally changed.

Stage 5: Twenty post-pancreatectomy patients were interviewed to assess wording, relevance, and comprehensibility. Participants reported no ambiguity or incomprehension. Therefore, the prefinal version was accepted without further item deletion or conceptual modification as the final Chinese PEI-Q.

### 2.5. Participants and data collection

Between February and October 2021, participants were recruited from the outpatient department of the First Affiliated Hospital with Nanjing Medical University among patients attending follow-up visits after pancreatic surgery. Convenience sampling, rather than consecutive sampling, was used. Inclusion criteria were native Chinese speakers aged 18 to 70 years, a history of pancreatectomy and long-term pancreatin use according to the surgeon’s advice, ability to understand the questionnaire, and provision of written informed consent. Patients were excluded if they could not understand or complete the questionnaire for any reason. The available analysis database contained 163 questionnaire records.

The age range of 18 to 70 years was selected for this initial validation study to include adult postoperative patients while reducing the potential influence of severe age-related cognitive or functional limitations on questionnaire completion. This criterion means that the present findings should not be generalized to older post-pancreatectomy patients without further validation.

### 2.6. Sample size

The sample size was calculated according to the commonly used recommendation that psychometric studies include 5 to 10 participants per scale item.^[25]^ Because the PEI-Q contains 18 items, the preferred sample size was 90 to 180 participants. However, simulation evidence shows that confirmatory factor analysis (CFA) sample-size requirements vary with factor loadings, model complexity, missing data, and estimator characteristics, so item-to-participant rules should be regarded as rough planning guides rather than guarantees of adequate power.^[26]^ The final available analysis sample (n = 163) met the lower recommended requirement and was close to the preferred 10 participants per item. However, this sample size is marginal for conducting both exploratory factor analysis and CFA, and the CFA results should be interpreted as preliminary because they were obtained from the same dataset used for exploratory analysis.

### 2.7. Missing data and response rate

The PEI-Q item-level data were reviewed before analysis. One blank item response was identified; because the recorded impact-domain total was equal to the sum of the other 4 impact items, the blank was coded as 0 for scoring consistency. No other PEI-Q item-level missing values were present. Demographic and clinical variables had variable missingness and were summarized using available-case denominators. Missingness was concentrated in retrospectively recorded clinical variables rather than PEI-Q items, which is compatible with incomplete record documentation; however, the missingness mechanism could not be tested and should not be assumed to be completely at random. Because these variables were descriptive and were not required for the primary item-level psychometric analyses, no imputation was used. Missingness percentages were reported separately to improve transparency. Two negative postoperative intervals caused by inconsistent dates were treated as missing for postoperative interval calculations. One implausible current-weight entry that produced an impossible body mass index (BMI) value was also treated as missing for current BMI calculation.

### 2.8. Statistical analysis

Data were double-checked and entered into Excel, and analyses were performed using IBM SPSS Statistics 26 and independent statistical verification in Python. Continuous variables are presented as mean ± standard deviation, and categorical variables are presented as frequencies and percentages. Available-case denominators and missingness percentages were reported for demographic and clinical variables with missing values.

Item analysis was performed using the critical ratio (CR), Pearson item-total correlations, corrected item-total correlations, and Cronbach alpha if item deleted.^[27]^ Corrected item-total correlations below 0.30 were treated as potentially weak screening signals, not as automatic deletion criteria. Shapiro–Wilk tests indicated that total and domain scores were non-normally distributed; therefore, CR findings were interpreted cautiously as conventional psychometric screening indices rather than definitive evidence of item performance.

Construct validity was assessed using principal component analysis (PCA)-based exploratory analysis with varimax rotation, CFA of the original 3-domain structure, and principal axis factoring (PAF) sensitivity analysis with varimax rotation.^[28,29]^ PCA was retained for comparability with the earlier PEI-Q analysis and to describe empirical component patterns in a small single-center dataset. PCA and PAF used the Pearson item-correlation matrix. However, PCA is a data-reduction technique rather than a latent variable method; therefore, no latent factor claims were based on PCA alone. PAF was performed as a sensitivity analysis because it is a common factor extraction method and is more consistent with latent construct evaluation. Varimax was selected solely to maintain comparability with the prior PEI-Q analysis; it was not intended to imply that the symptom domains are uncorrelated. Because the domains may be correlated, an oblique rotation should be evaluated in a larger independent sample. The 5 response categories are ordinal, but conventional CFA was used here as a preliminary comparability analysis rather than as a definitive categorical-data model. Ordinal CFA with an estimator appropriate for categorical indicators should be prioritized in future validation work.^[30]^ Parallel analysis used the Pearson item-correlation matrix and 1000 random normal datasets with the same sample size and number of items; factors were retained when the observed eigenvalue exceeded the simulated 95th-percentile eigenvalue.^[31]^ This procedure retained 3 factors. Because PCA, PAF, and CFA were conducted in the same dataset, all structural findings were interpreted as preliminary and descriptive.

Distributional performance was evaluated using floor and ceiling effects for the abdominal symptoms domain, bowel movement symptoms domain, total symptom score, impacts domain, and total summary score. Floor effects were defined as the percentage of participants with the minimum possible score, and ceiling effects were defined as the percentage with the maximum possible score.

Reliability was evaluated using internal consistency, and Cronbach alpha coefficients with bootstrap 95% confidence intervals (CIs) were calculated for the total scale and each domain.^[32]^ Cronbach alpha evaluates internal consistency and does not establish temporal stability or overall reliability. McDonald omega was estimated as complementary internal-consistency evidence using a 1-factor principal-axis model fitted to the item covariance matrix.^[33]^ The omega estimates are model-based and should not be interpreted as evidence of temporal stability. Content validity was evaluated by 7 experts from the Pancreas Center of Jiangsu Province using the item-level content validity index (I-CVI) and the average scale-level content validity index (S-CVI/Ave).^[34–39]^ Because the available database did not contain a complete prespecified retest subset, intraclass correlation coefficient (ICC)-based test–retest reliability could not be estimated.

## 3. Results

### 3.1. Participant flow and demographic characteristics

**Figure 1.**
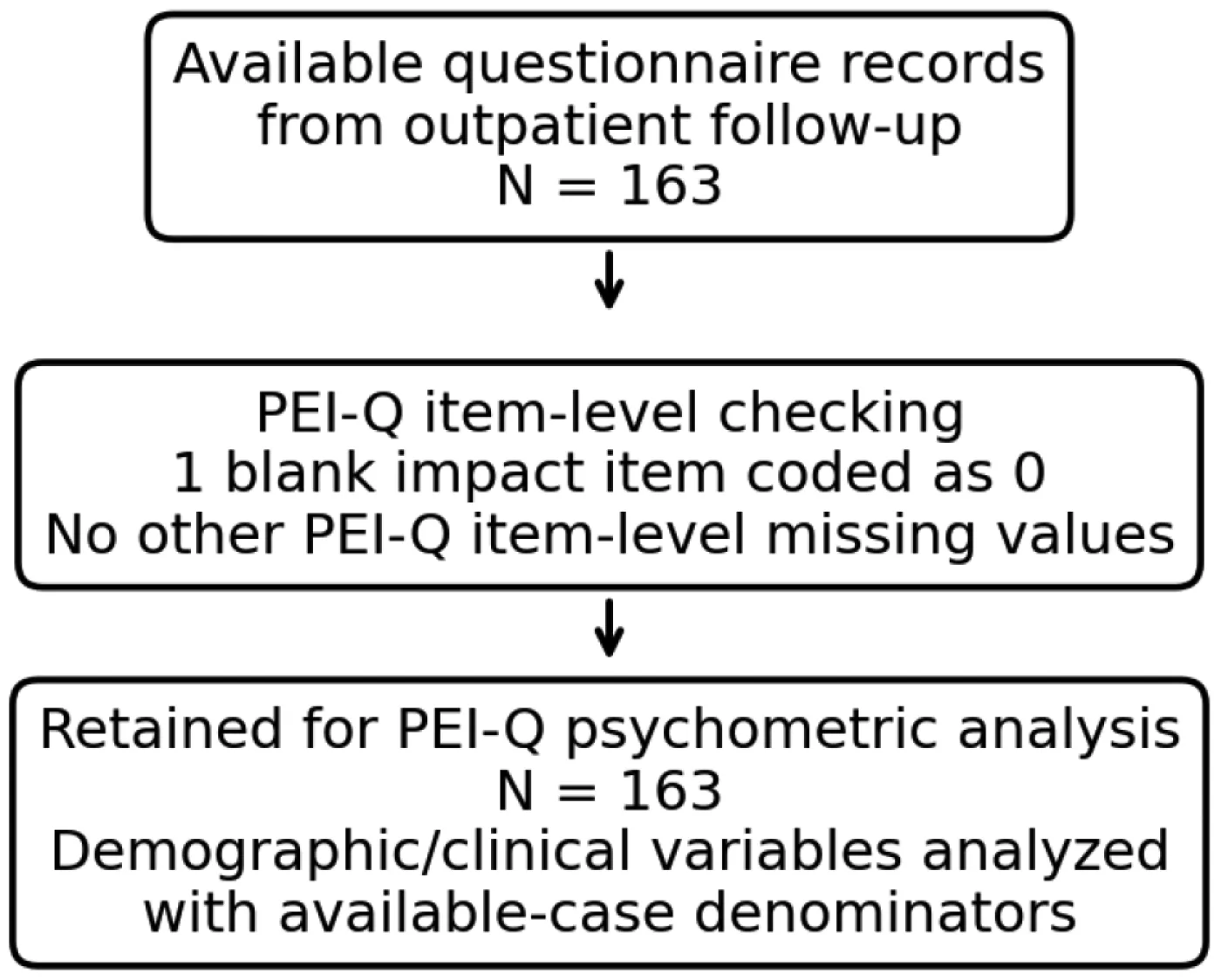
Participant flow diagram. PEI-Q = Pancreatic Exocrine Insufficiency Questionnaire.

The available database contained 163 questionnaire records. After item-level checking, all 163 records were retained for PEI-Q psychometric analysis. Demographic and clinical variables had different amounts of missing data, so valid denominators and missingness percentages are shown in Tables 2 and 3. The participant flow is shown in Figure 1.

**Table 2:** Demographic and clinical characteristics of participants (available analysis sample, N = 163).

Characteristic	Mean ± SD or n (%)	Valid n	Missing n (%)
Age, yr	60.75 ± 9.41	130	33 (20.2%)
Gender	Female: 79 (51.3) Male: 75 (48.7)	154	9 (5.5%)
Marital status	Married: 117 (91.4) Widowed: 10 (7.8) Divorced: 1 (0.8)	128	35 (21.5%)
Education level	Junior high school or below: 63 (50.0) High school: 33 (26.2) Junior college: 16 (12.7) Undergraduate: 11 (8.7) Postgraduate: 3 (2.4)	126	37 (22.7%)
Medical payment	Health insurance: 118 (94.4) Out of pocket: 6 (4.8) Public expense: 1 (0.8)	125	38 (23.3%)
Residence type	Urban: 94 (74.6) Rural: 32 (25.4)	126	37 (22.7%)
Admission BMI, kg/m^2^	23.42 ± 3.36	119	44 (27.0%)
Current BMI, kg/m^2^	22.28 ± 3.31	111	52 (31.9%)
Postoperative interval, days	185.40 ± 278.25	95	68 (41.7%)
Type of operation	Pancreatoduodenectomy/PPPD/LPD: 55 (53.4) Distal pancreatectomy: 35 (34.0) Exploratory/palliative or other procedure: 9 (8.7) Other pancreatic operation: 3 (2.9) Total pancreatectomy: 1 (1.0)	103	60 (36.8%)

Valid n is the number of observed values. Missing n (%) is calculated against the full available analysis sample (N = 163); percentages within categorical variables are based on valid observations.

BMI = body mass index, LPD = laparoscopic pancreatoduodenectomy, PPPD = pylorus-preserving pancreatoduodenectomy, SD = standard deviation.

**Table 3:** Missingness summary for demographic and clinical variables (N = 163).

Variable	Valid n	Missing n	Missing (%)
PEI-Q item-level analysis after 1 blank response was coded as 0	163	0	0.0
Age	130	33	20.2
Gender	154	9	5.5
Marital status	128	35	21.5
Education level	126	37	22.7
Medical payment	125	38	23.3
Residence type	126	37	22.7
Admission BMI	119	44	27.0
Current BMI	111	52	31.9
Postoperative interval	95	68	41.7
Type of operation	103	60	36.8

BMI = body mass index, PEI-Q = Pancreatic Exocrine Insufficiency Questionnaire.

### 3.2. Item analysis

The CR values ranged from 2.547 to 9.090. All CR tests were statistically significant (*P* < .05), although item 12 showed weaker discrimination than most other items. Corrected item-total correlations ranged from −0.006 to 0.680; values below 0.30 were treated as potentially weak screening signals rather than automatic deletion criteria. Item 14 showed a very weak corrected item-total correlation (−0.006), and deleting this item increased the total alpha from 0.813 to 0.843. The item was retained to preserve the original PEI-Q content and scoring structure, but this finding indicates that item 14 requires further evaluation and possible cultural refinement or replacement (Table 4).

**Table 4:** Item analysis results.

Item	Domain	CR (*P* value)	Corrected item-total *r*	Alpha if item deleted
1	Abdominal symptoms	3.428 (.001)	0.277	0.811
2	Abdominal symptoms	7.419 (<.001)	0.422	0.803
3	Abdominal symptoms	6.706 (<.001)	0.475	0.800
4	Abdominal symptoms	6.176 (<.001)	0.292	0.812
5	Abdominal symptoms	5.889 (<.001)	0.301	0.812
6	Abdominal symptoms	5.979 (<.001)	0.556	0.794
7	Abdominal symptoms	7.757 (<.001)	0.487	0.799
8	Bowel movement symptoms	4.030 (<.001)	0.402	0.804
9	Bowel movement symptoms	3.661 (<.001)	0.431	0.803
10	Bowel movement symptoms	5.312 (<.001)	0.452	0.802
11	Bowel movement symptoms	6.099 (<.001)	0.357	0.807
12	Bowel movement symptoms	2.547 (.014)	0.439	0.804
13	Bowel movement symptoms	3.925 (<.001)	0.566	0.801
14	Impacts	3.584 (<.001)	-0.006	0.843
15	Impacts	9.090 (<.001)	0.608	0.790
16	Impacts	3.275 (.002)	0.568	0.801
17	Impacts	4.733 (<.001)	0.680	0.792
18	Impacts	4.273 (<.001)	0.552	0.800

Corrected item-total correlation refers to the correlation between each item and the total score after removing that item from the total. Values below 0.30 were treated as potentially weak screening signals, not as automatic deletion criteria.

CR = critical ratio.

### 3.3. Content validity

The 7 experts included 4 surgeons with doctoral degrees and >15 years of working experience, 2 head nurses with >20 years of working experience, and 1 statistician with a doctoral degree. The I-CVI values ranged from 0.857 to 1.000, and the S-CVI/Ave was 0.968. No item was deleted or conceptually modified after expert consultation.

### 3.4. Construct validity

The Kaiser–Meyer–Olkin (KMO) value was 0.818, and Bartlett test of sphericity was significant (chi-square = 1334.481, degrees of freedom = 153, *P* < .001), indicating that the data were suitable for exploratory analysis. At the item level, the varimax-rotated PAF sensitivity solution showed cross-loadings and a near-zero communality for item 14; the complete loading matrix is presented in Table 5. The scree plot suggested a dominant 1st component and possible retention of 3 to 4 components, and the 4th eigenvalue was also >1.00 (1.206). Parallel analysis based on 1000 random normal datasets retained 3 factors; the 4th observed eigenvalue was below the simulated 95th-percentile value (1.378)^[31]^ (Table 6). The 3-component solution was therefore retained for descriptive comparability with the predefined 3-domain PEI-Q structure while recognizing that empirical factor retention does not confirm the original domain structure.

**Table 5:** Varimax-rotated principal-axis factor loadings and communalities of the Chinese PEI-Q.

Item	Conceptual domain	PAF Factor 1	PAF Factor 2	PAF Factor 3	Communality
1	Abdominal symptoms	0.410	0.170	−0.112	0.209
2	Abdominal symptoms	0.185	0.163	0.508	0.319
3	Abdominal symptoms	0.670	0.059	0.046	0.455
4	Abdominal symptoms	0.016	0.036	0.736	0.543
5	Abdominal symptoms	0.030	−0.011	0.818	0.671
6	Abdominal symptoms	0.686	0.226	0.006	0.522
7	Abdominal symptoms	0.705	0.099	−0.017	0.507
8	Bowel movement symptoms	0.167	0.644	0.003	0.442
9	Bowel movement symptoms	0.117	0.815	−0.003	0.678
10	Bowel movement symptoms	0.239	0.438	0.175	0.280
11	Bowel movement symptoms	0.019	0.085	0.816	0.673
12	Bowel movement symptoms	0.224	0.554	0.134	0.375
13	Bowel movement symptoms	0.243	0.785	0.100	0.686
14	Impacts	0.068	−0.093	−0.011	0.013
15	Impacts	0.582	0.108	0.338	0.464
16	Impacts	0.514	0.559	0.020	0.578
17	Impacts	0.686	0.424	0.138	0.669
18	Impacts	0.707	0.300	−0.034	0.590

Loadings are principal axis factoring (PAF) coefficients after varimax rotation; values were not suppressed. The matrix is a sensitivity analysis and does not confirm the original 3-domain structure. Item 14 had the lowest communality (0.013).

PEI-Q = Pancreatic Exocrine Insufficiency Questionnaire.

**Table 6:** PCA eigenvalues and parallel-analysis results.

Component	Observed eigenvalue	Variance explained (%)	Mean random eigenvalue	95th-percentile random eigenvalue	Retained?
Component 1	5.650	31.39	1.627	1.744	Yes
Component 2	2.597	14.43	1.499	1.589	Yes
Component 3	1.788	9.94	1.404	1.481	Yes
Component 4	1.206	6.70	1.319	1.378	No
Component 5	0.959	5.33	1.245	1.301	No
Component 6	0.831	4.62	1.179	1.233	No
Component 7	0.709	3.94	1.115	1.161	No
Component 8	0.688	3.82	1.055	1.102	No
Component 9	0.623	3.46	0.997	1.045	No
Component 10	0.509	2.83	0.940	0.987	No
Component 11	0.440	2.44	0.888	0.936	No
Component 12	0.418	2.32	0.835	0.879	No
Component 13	0.397	2.20	0.785	0.829	No
Component 14	0.340	1.89	0.731	0.774	No
Component 15	0.260	1.45	0.681	0.729	No
Component 16	0.215	1.19	0.627	0.671	No
Component 17	0.195	1.08	0.571	0.623	No
Component 18	0.175	0.97	0.502	0.566	No

Parallel analysis used the Pearson item-correlation matrix and 1000 random normal datasets with n = 163 and *P* = 18 (seed = 20,260,717); factors were retained when the observed eigenvalue exceeded the simulated 95th-percentile eigenvalue. Three factors were retained. The 4th observed eigenvalue was 1.206, below the simulated 95th-percentile value of 1.378.

PCA = principal component analysis.

The 3-component PCA solution explained 55.75% of the total variance (Table 6). PCA communalities ranged from 0.050 to 0.728. Item 14 had the lowest PCA communality (0.050) and did not load meaningfully on the retained components. CFA of the original 3-domain structure showed poor fit (chi-square = 491.704, degrees of freedom = 132, comparative fit index = 0.710, Tucker–Lewis index = 0.664, root mean square error of approximation = 0.130, and standardized root mean square residual = 0.130). These fit indices fall below commonly accepted thresholds and indicate that the hypothesized original 3-domain structure was not supported in this sample. PAF sensitivity analysis produced a similar cautionary pattern. The 3-factor PAF solution had rotated sums of squared loadings of 3.405, 2.926, and 2.343, corresponding to 18.92%, 16.25%, and 13.02% of variance, respectively, with a cumulative value of 48.19%. The varimax-rotated PAF loading matrix is presented in Table 5. Item 14 again showed the weakest performance, with a PAF communality of 0.013 and no meaningful loading on any retained factor. The PAF sensitivity analysis did not resolve the structural-validity concern, despite the 3-factor retention indicated by parallel analysis (Table 6).

### 3.5. Distributional floor and ceiling effects

Floor and ceiling effects were calculated from the individual-level item scores (Table 7). The floor effect was 4.3% for abdominal symptoms, 25.8% for bowel movement symptoms, 3.7% for the total symptom score, 9.2% for impacts, and 0.0% for the total summary score. No ceiling effect was observed for any domain or summary score. The 25.8% floor effect means that approximately 1 in 4 participants received the minimum possible bowel movement symptom score. This may limit discrimination among patients with few or no bowel-related symptoms, although it may also reflect the case mix and treatment context of this postoperative sample rather than an intrinsic lack of clinical relevance. The higher floor effect should therefore be considered when interpreting the domain and when evaluating responsiveness in future samples.

**Table 7:** Floor and ceiling effects of the Chinese PEI-Q.

Scale/domain	Minimum score definition	Floor effect, n (%)	Maximum score definition	Ceiling effect, n (%)
Abdominal symptoms	A = 0	7 (4.3%)	A = 4	0 (0.0%)
Bowel movement symptoms	B = 0	42 (25.8%)	B = 4	0 (0.0%)
Total symptom score	(A + B)/2 = 0	6 (3.7%)	(A + B)/2 = 4	0 (0.0%)
Impacts	C = 0	15 (9.2%)	C = 4	0 (0.0%)
Total summary score	(A + B + C)/3 = 0	0 (0.0%)	(A + B + C)/3 = 4	0 (0.0%)

Floor and ceiling effects were calculated among all 163 participants using individual-level item scores. All scores have a possible range of 0 to 4.

PEI-Q = Pancreatic Exocrine Insufficiency Questionnaire.

### 3.6. Reliability

The overall Cronbach alpha was 0.813 (95% CI: 0.742–0.857). Cronbach alpha values for the abdominal symptoms, bowel movement symptoms, and impacts domains were 0.667, 0.719, and 0.603, respectively (Table 8). The abdominal symptoms and impacts domains were below the conventional 0.70 threshold and should therefore be interpreted as borderline in this dataset. McDonald omega values were 0.820 for the total scale and 0.653, 0.733, and 0.639 for the abdominal symptoms, bowel movement symptoms, and impacts domains, respectively. Cronbach alpha and omega provide evidence about internal consistency only; neither establishes temporal stability or overall reliability. Test–retest reliability was not available, so temporal stability remains unknown.

**Table 8:** Internal consistency of the Chinese PEI-Q.

Scale/domain	Cronbach alpha	95% CI	McDonald omega	Interpretation
Total PEI-Q	0.813	0.742–0.857	0.820	Acceptable
Abdominal symptoms	0.667	0.580–0.735	0.653	Borderline
Bowel movement symptoms	0.719	0.595–0.792	0.733	Acceptable
Impacts	0.603	0.465–0.699	0.639	Borderline

Cronbach alpha evaluates internal consistency and does not establish temporal stability or overall reliability. McDonald omega was estimated as omega-total from a 1-factor principal-axis factor model fitted to the item covariance matrix; it is complementary model-based evidence of internal consistency and does not establish temporal stability.

CI = confidence interval, PEI-Q = Pancreatic Exocrine Insufficiency Questionnaire.

## 4. Discussion

This study translated and cross-culturally adapted the PEI-Q into simplified Chinese and evaluated selected psychometric properties among 163 available post-pancreatectomy questionnaire records. The Chinese version retained all 18 translated items and showed strong content validity and acceptable overall internal consistency. However, the CFA results indicate that the original 3-domain structure was not confirmed in this sample. Therefore, construct validity remains preliminary, and the Chinese PEI-Q should be regarded as an adjunctive symptom assessment instrument under development rather than a fully validated measurement or diagnostic tool.

Item analysis identified item 14 as the main psychometric concern. Although all CR tests were statistically significant, item 14 had a corrected item-total correlation close to zero, the lowest communality, and an increase in total alpha if deleted. Item 14 assesses avoidance of fatty foods. In the Chinese postoperative context, this behavior may not reflect PEI-related impact alone. It may instead reflect physician or nurse dietary counseling, routine low-fat diet advice after pancreatic surgery, personal eating habits, regional differences in oil-rich foods, fear of postoperative symptoms, family dietary control, or the wording used to express fatty-food avoidance in Chinese. Therefore, item 14 may be measuring a health behavior or culturally shaped dietary response rather than the intended impact construct.

Although item 14 was retained for comparability with the original PEI-Q and to preserve content coverage at this initial validation stage, its current performance is not satisfactory. Future Chinese PEI-Q studies should conduct cognitive interviewing focused on this item, compare alternative Chinese wording, examine whether responses differ by dietary counseling and pancreatic enzyme replacement therapy, and consider revising or replacing the item if poor item-total correlation and low communality persist in independent samples.

The overall Cronbach alpha of 0.813 indicates acceptable internal consistency for the total scale. In contrast, the abdominal symptoms domain and impacts domain were below the conventional 0.70 threshold in the available dataset. These lower values may reflect heterogeneous postoperative symptom experiences, the small number of items within each domain, cultural differences in reporting symptom impacts, and the influence of operation type, postoperative interval, enzyme replacement, and diet. Domain scores should therefore be interpreted cautiously.

Reliability evidence remains incomplete because only internal consistency was evaluated. Test–retest reliability could not be estimated using ICC because the available analysis database did not contain a complete prespecified retest subset. Temporal stability is therefore unknown. Future validation studies should prioritize paired retest data collection and report ICCs with 95% CIs for the total scale and domains.

Content validity findings were strong. The expert panel included surgeons, senior nurses, and a statistician from a pancreatic center, and the I-CVI and S-CVI/Ave values met commonly accepted standards.^[34,35]^ During cross-cultural adaptation, wording related to stool characteristics, bowel movement frequency, and fatty food avoidance was reviewed carefully because these concepts may be expressed differently across educational and regional groups. Nevertheless, strong content validity does not offset the need for further structural, temporal, criterion, and responsiveness evidence.

Construct validity findings were weak and should be interpreted cautiously. The Kaiser–Meyer–Olkin and Bartlett results supported exploratory analysis, and the 3 retained PCA components explained 55.75% of variance. However, PCA is not a latent variable method; item allocation was not identical to the original English version; the 4th eigenvalue was above 1.00, item 14 did not contribute meaningfully; and CFA showed poor fit.^[40,41]^ Parallel analysis retained 3 factors, and the PAF pattern matrix showed the item-level coefficients for that descriptive sensitivity solution, but neither result confirmed the original 3-domain structure. These results do not establish whether the poor fit reflects item wording, cross-loadings, response distributions, postoperative heterogeneity, or a different structure. No alternative latent dimensions should be inferred without independent data and an appropriate ordinal common-factor analysis. The original 3-domain structure should therefore be considered unconfirmed until replicated in larger independent samples.

This difference reinforces the need for independent cross-validation before the translated scale is treated as structurally stable in Chinese-speaking postoperative populations.^[18,42]^

The present study also does not establish criterion validity or diagnostic accuracy. The questionnaire was not compared with fecal elastase-1, fecal fat excretion, clinical diagnosis, or another validated PEI measure. As a result, higher PEI-Q scores in this study should be interpreted as greater self-reported symptom or impact burden, not as evidence of clinically confirmed PEI. Future studies should evaluate sensitivity, specificity, predictive values, clinically meaningful cutoff scores, and agreement with biochemical and clinical standards.

Responsiveness was not evaluated. Because PEI-related symptoms may change after pancreatic enzyme replacement, nutritional intervention, postoperative recovery, or disease progression, future longitudinal studies should determine whether the Chinese PEI-Q can detect clinically meaningful change over time. Responsiveness evidence will be important before the instrument can be used to monitor treatment response.

### 4.1. Strengths and limitations

This study has strengths. It used a standardized cross-cultural adaptation framework, obtained authorization from the original author, included multidisciplinary expert review, performed pilot testing, added corrected item-total correlations, reported communalities and eigenvalues, and attempted CFA of the original structure. It also used available clinical variables to describe the postoperative sample more transparently and clearly separated symptom-assessment evidence from diagnostic validity.

First, this was a single-center study using convenience sampling, and demographic and clinical variables had missing values. Selection bias, socioeconomic bias, and regional linguistic bias are possible. Because recruitment occurred among patients returning for outpatient follow-up, participants may have been healthier, more adherent, or more engaged with care than eligible patients who did not return, creating an additional source of selection bias. The findings should not be generalized to all Chinese patients after pancreatectomy without multicenter validation.

Second, structural validity remains preliminary. The original 3-domain structure was not confirmed by CFA, and PCA-based exploratory results should not be interpreted as latent factor confirmation. Although PAF pattern coefficients and parallel-analysis results were added, the analysis did not include ordinal CFA or an oblique-rotation solution. Larger independent datasets are required, ideally using ordinal CFA, common-factor methods, oblique rotation, and empirically supported factor-retention procedures appropriate for Likert-type data.

Third, both exploratory analysis and CFA were conducted on the same sample of 163 participants. Although the sample size met a minimum participant-to-item recommendation, it is marginal for simultaneous exploratory factor analysis and CFA. Future studies should use 1 sample for exploratory analysis and an independent sample for CFA.

Fourth, criterion validity and diagnostic accuracy were not evaluated against fecal elastase-1, fecal fat excretion, clinical diagnosis, or other biochemical or clinical standards. The Chinese PEI-Q should not be interpreted as a stand-alone diagnostic test for PEI.

Fifth, several important postoperative clinical variables were either unavailable or incompletely recorded, including pancreatic enzyme dosage, nutritional status, tumor stage, diabetes status, and postoperative complications. Missingness limited further analysis of their influence on questionnaire responses.

Sixth, ICC-based test–retest reliability could not be reported because the available analysis database did not contain a complete prespecified retest subset. Temporal stability of the Chinese PEI-Q remains unknown.

Seventh, floor and ceiling effects were quantified in this dataset, and the bowel movement symptoms domain showed a notable floor effect of 25.8%. Although no ceiling effects were observed, this floor effect may reduce sensitivity among patients with mild or absent bowel-related symptoms. Responsiveness was not evaluated because longitudinal follow-up after treatment was unavailable. Future studies should determine whether the Chinese PEI-Q can detect clinically meaningful changes after pancreatic enzyme replacement therapy, nutritional intervention, or postoperative recovery.

Eighth, participants older than 70 years were not included. Because older postoperative patients may have a high risk of PEI and unique difficulties with questionnaire completion, the Chinese PEI-Q should be further evaluated in older age groups.

Finally, 1 item-level blank response was coded as 0 based on the recorded domain total, and demographic/clinical variables were analyzed using available cases. Although this approach preserved the PEI-Q item analysis sample, missing demographic and clinical information may still introduce bias. McDonald omega was estimated as model-based internal-consistency evidence, but it does not address temporal stability; ICC-based test–retest reliability, responsiveness, and criterion validity remain priorities for future validation.

## 5. Conclusion

The Chinese PEI-Q showed acceptable overall internal consistency and strong content validity in this single-center post-pancreatectomy sample. However, the original 3-domain structure was not confirmed, item 14 showed serious psychometric weakness, test-retest reliability was unavailable, and criterion validity, diagnostic accuracy, and responsiveness were not established. Floor and ceiling effects were quantified; no ceiling effects were observed, but the bowel movement symptoms domain showed a notable floor effect. The instrument may be useful for PEI-related symptom assessment and patient-provider communication in postoperative pancreatic care. It should not be used as a stand-alone diagnostic tool or enter routine clinical implementation as a fully validated Chinese PEI measure until further multicenter validation, criterion-validity testing, temporal-stability assessment, and responsiveness evaluation are completed.

## Acknowledgments

We thank Dr Colin D. Johnson for permission to translate and cross-culturally adapt the PEI-Q. We are grateful to Drs Chuhui Zeng and Min Lin for assistance with back translation and to Dr Kai Zhang (The First Affiliated Hospital with Nanjing Medical University) for guidance on data analysis. All individuals named above gave permission to be identified in this section.

## Author contributions

**Data curation:** Wang Su, Lei Cui, Jianjian Wang, Kuirong Jiang, Xiaoping Fang.

**Writing – original draft:** Huiping Yu.

**Writing – review & editing:** Huiping Yu.
